# Supplementary material for: Classifier uncertainty: evidence, potential impact, and probabilistic treatment
Source: PeerJ Comput Sci. 2021 Mar 4;7:e398. doi: 10.7717/peerj-cs.398 (PMC7959610; doi:10.7717/peerj-cs.398)
Supplement: Supplemental Information 1 [file peerj-cs-07-398-s001.pdf]

Supporting Information for Classifier uncertainty: evidence, potential impact, and probabilistic treatment

Niklas Tötsch[1] and Daniel Hoffmann[1]

[1]**Faculty of Biology, University of Duisburg-Essen, Essen, Germany**

Corresponding author:
Niklas Tötsch[1]

Email address: niklas.toetsch@uni-due.de

PRIORS

The prior distribution can be interpreted as expression of previous knowledge, which in turn can be expressed in terms of previous observations. In this sense, the Laplace (or flat) prior is equivalent to two previous observations for each prevalence (ϕ), true positive rate (TPR) and true negative rate (TNR), which is usually a questionable assumption. Since sample size (N) is small in some of the examples discussed in this study, this assumption could have an impact on the posterior distribution. Nevertheless, we consider this prior to be the most suitable objective prior. Haldane's prior, $\mathrm{Beta}(\alpha = 0, \beta = 0)$, is not adequate since it yields an improper posterior if any entry of the confusion matrix (CM) is zero, which is often the case. Jeffreys prior, $\mathrm{Beta}(\alpha = 0.5, \beta = 0.5)$, does not have this problem but leads to implausible U-shaped priors for some metrics (Figure S2).

MARGINALS OF THE CONFUSION MATRIX

There are three scenarios for the marginals of the CM. In principle, the marginals of the columns and rows of the CM could both be fixed, which would mean that ϕ and the number of positive/negative predictions are known exactly beforehand. Fisher's exact test was designed to evaluate whether a binary classifier performs better than random guessing for this specific case. Fisher (1922) It remains popular, yet the underlying assumption is usually violated. McElreath (2018); Gelman (2003)

A fixed ϕ and an unspecified marginal on the predicted labels is more common. For instance, in a controlled study, test sets may be curated to include 50% patients suffering from a disease and 50% healthy subjects in a control group. In this example there is no uncertainty in ϕ, but it is fixed at ϕ=0.5.

If ϕ in the test set is not deliberately chosen before the compilation, it must be determined from the data set. For small sample sizes, ϕ is uncertain like all other metrics. In the present study, we infer ϕ from the CM but our method also copes with fixed ϕ.

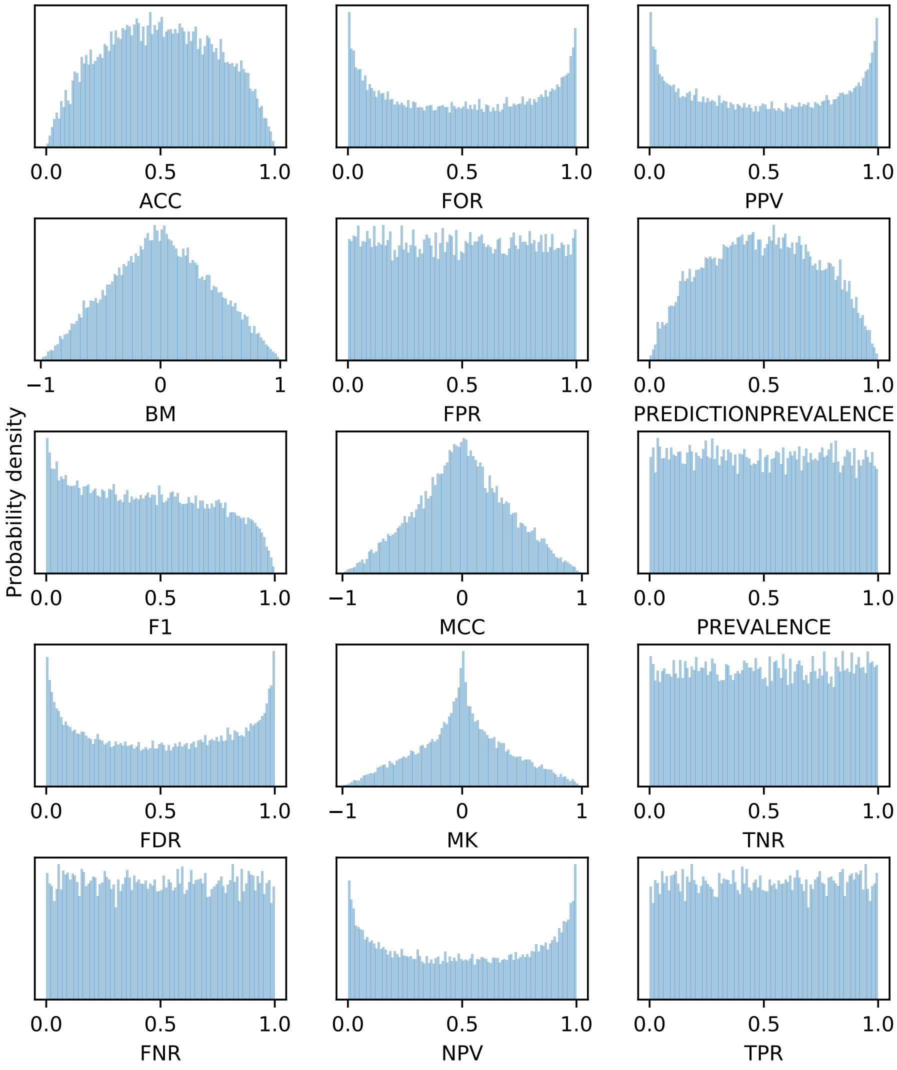

Figure S1. Priors on the metrics if Laplace priors are used for ϕ, TPR, TNR

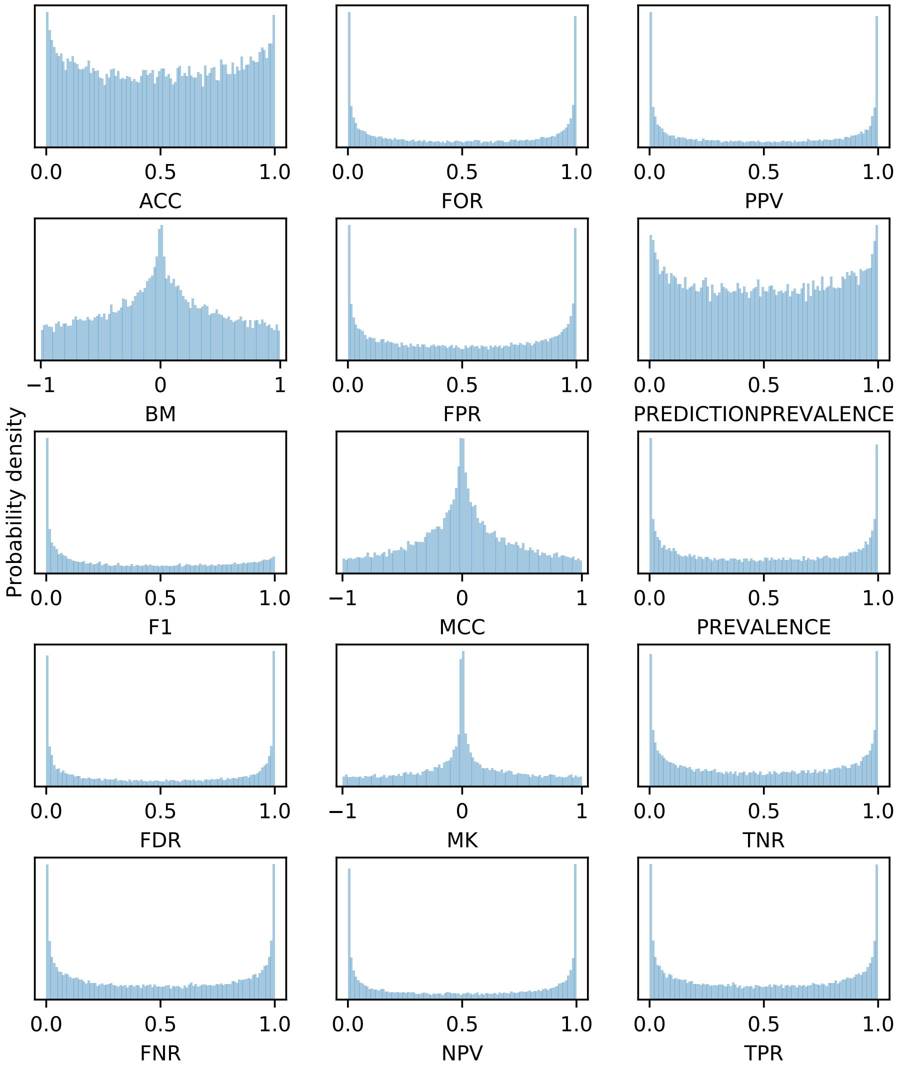

Figure S2. Priors on the metrics if Jeffreys priors are used for ϕ, TPR, TNR

LITERATURE EXAMPLES

Table S1. Literature examples of classifiers with small sample size (N). Citations were recorded on Google Scholar on June 16th, 2020 at 12:55 pm CEST.

	DOI	Location	TP	FN	TN	FP	N	Citations
1	10.1080/10629360903278800	Table 2	5	0	3	0	8	10
2	10.1021/ci200579f	Table 3	10	0	3	1	14	48
3	10.1021/ci020045	Table 5	6	0	7	1	14	51
4a	10.1155/2015/485864	Table 4	5	1	10	1	17	10
4b	10.1155/2015/485864	Table 5	4	2	10	1	17	10
5a	10.1016/j.ejmech.2010.11.029	Table 6	16	1	3	2	22	86
5b	10.1016/j.ejmech.2010.11.029	Table 10	8	9	4	1	22	86
6a	10.1016/j.vascn.2014.07.002	Table 2	2	12	19	1	34	77
6b	10.1016/j.vascn.2014.07.002	Table 3	10	4	20	0	34	77
7a	10.5935/0103-5053.20130066	Table 2	26	0	6	2	34	61
7b	10.5935/0103-5053.20130066	Table 3	24	2	6	2	34	61
8	10.1016/j.scitotenv.2018.05.081	Table 2	28	9	3	4	44	18
9a	10.4314/wsa.v36i4.58411	Table 2	19	3	18	10	50	14
9b	10.4314/wsa.v36i4.58411	Table 2	21	1	20	8	50	14
10	10.1016/j.bspc.2017.01.012	Figure 2	31	5	24	4	64	80
11	10.1039/C7MD00633K	Figure 3	40	7	15	8	70	9
12	10.3389/fnins.2018.01008	Figure 3	31	9	20	13	73	1
13a	10.4315/0362-028X-61.2.221	Table 3	79	14	19	0	112	52
13b	10.4315/0362-028X-61.2.221	Table 3	89	4	16	3	112	52
14a	10.1016/j.ancr.2014.06.005	Figure 6.3	136	2	2	12	152	7
15a	10.1016/j.saa.2016.09.028	Table 2	3	12	150	0	165	65
15b	10.1016/j.saa.2016.09.028	Table 2	6	9	150	0	165	65
16	10.1021/acs.analchem.7b00426	Table 3	188	0	13	2	203	28
14b	10.1016/j.ancr.2014.06.005	Table 3	253	27	11	59	350	7

PROOF THAT VARIANCE OF METRIC DISTRIBUTIONS CALCULATED FROM SYNTHETIC CONFUSION MATRICES IS SYSTEMATICALLY TOO LARGE

For a confusion probability matrix (θ) following a Dirichlet distribution with parameter vector α

$$\theta \sim \text{Dirichlet}(\alpha) \tag{S1}$$

where α is the sum of the CM and the prior, the expected value and variance are

$$\mathrm{E}\left[\theta_i\right] = \frac{\alpha_i}{\alpha_0} \tag{S2}$$

$$\mathrm{Var}\left[\theta_i\right] = \frac{\alpha_i}{\alpha_0}\left(\frac{1-\frac{\alpha_i}{\alpha_0}}{1+\alpha_0}\right) \tag{S3}$$

where $\alpha_0 = \sum \alpha_k$. The expected value and variance of the entry V_i of a confusion matrix generated by a multinomial distribution

$$V = [V_{\mathrm{TP}}, V_{\mathrm{FN}}, V_{\mathrm{TN}}, V_{\mathrm{FP}}] \sim \text{Multinomial}(\theta, N) \tag{S4}$$

is given by

$$\mathrm{E}\left[V_i\right] = N\frac{\alpha_i}{\alpha_0} = N\mathrm{E}\left[\theta_i\right] \tag{S5}$$

$$\mathrm{Var}\left[V_i\right] = N(N+\alpha_0)\frac{\alpha_i}{\alpha_0}\left(\frac{1-\frac{\alpha_i}{\alpha_0}}{1+\alpha_0}\right) = N(N+\alpha_0)\mathrm{Var}\left[\theta_i\right] \tag{S6}$$

From this, we can calculate the expected value and variance for the proportion of i, $\frac{V_i}{N}$

$$\mathrm{E}\left[\frac{V_i}{N}\right] = \frac{1}{N}\mathrm{E}\left[V_i\right] = \mathrm{E}\left[\theta_i\right] \tag{S7}$$

$$\mathrm{Var}\left[\frac{V_i}{N}\right] = \frac{1}{N^2}\mathrm{Var}\left[V_i\right] = \left(1+\frac{\alpha_0}{N}\right)\mathrm{Var}\left[\theta_i\right] \tag{S8}$$

Whereas $\mathrm{E}\left[\frac{V_i}{N}\right]$ is independent of N, $\mathrm{Var}\left[\frac{V_i}{N}\right]$ is not. In Caelen's approach, $N \approx \alpha_0$. Therefore, the variance will be overestimated by approximately a factor of two. Since the variance of $\frac{V_i}{N}$ are overestimated w.r.t. θ_i, the same holds for $\frac{V}{N}$ w.r.t. θ and metrics calculated on $\frac{V}{N}$ and θ, respectively.

If N was increased beyond α_0, it would converge towards the true variance

$$\lim_{N\to\infty} \mathrm{Var}\left[\frac{V_i}{N}\right] = \mathrm{Var}\left[\theta_i\right]. \tag{S9}$$

RULE FOR SAMPLE SIZE DETERMINATION OF METRICS MODELED BY A BETA DISTRIBUTION

For a normal distribution, approximately 95% of the density are within two standard deviations σ from the mean. Therefore, the length of the 95% highest posterior density interval will be close to 4σ. According to the central limit theorem, beta distributions behave for large sample sizes like normal distributions. The standard deviation σ of a beta distribution is given by

$$\sigma = \sqrt{\frac{\alpha \cdot \beta}{(\alpha+\beta)^2(\alpha+\beta+1)}}. \tag{S10}$$

where α and β are the counts of observations per class, where the meaning of "class" depends on the studied metric. As discussed in the main text, if one is looking at accuracy (ACC), α denotes correct classifications (TP + TN) and β denotes wrong classifications (FP + FN). In the case of TPR, α counts the number of true positives (TPs) whereas β counts false negatives (FNs).

To make explicit the dependency on sample size N, we express α as $a \cdot N$ and β as $b \cdot N$ with fractions $a = \frac{\alpha}{N}, b = \frac{\beta}{N}$ of the two classes.

$$\sigma = \sqrt{\frac{a \cdot N \cdot b \cdot N}{(a \cdot N + b \cdot N)^2 (a \cdot N + b \cdot N + 1)}} \tag{S11}$$

$$\sigma = \sqrt{\frac{N^2 \cdot a \cdot b}{N^2 (a+b)^2 (N(a+b)+1)}} \tag{S12}$$

$$\sigma = \sqrt{\frac{a \cdot b}{(a+b)^2 (N(a+b)+1)}} \tag{S13}$$

Since $\alpha + \beta = N$, we know that $a + b = 1$. Now we can simplify Equation S13 to

$$\sigma = \sqrt{\frac{a \cdot b}{N+1}} \tag{S14}$$

For large N, this approximates to

$$\sigma \approx \sqrt{\frac{a \cdot b}{N}} \tag{S15}$$

σ is largest if $a = b = 0.5$.

$$\sigma_{max} \approx \sqrt{\frac{0.5 \cdot 0.5}{N}} \tag{S16}$$

$$\sigma_{max} \approx \frac{0.5}{\sqrt{N}} \tag{S17}$$

In the main text, we have defined metric uncertainty (MU) as the length of the 95% highest posterior density interval. Therefore, its upper limit can be approximated as $4\sigma \approx \frac{2}{\sqrt{N}}$. If one cannot reject the possibility that $a = b = 0.5$, one will need $\frac{4}{MU^2}$ samples to obtain the desired MU.

SIMULATED CLASSIFIER

We have simulated a classifier with known properties. ϕ is 50%, TPR equals 80%, and TNR is 60%. Bookmaker informedness (BM) is therefore 40%. We calculate θ_{TP}, θ_{FN}, θ_{TN}, and θ_{FP} as described in subsection 2.1. Confusion matrices of varying sizes are generated according to a multinomial distribution Mult($\theta_= \{\theta_{\text{TP}}, \theta_{\text{FN}}, \theta_{\text{TN}}, \theta_{\text{FP}}\}, N$). Posterior distributions of BM are determined as usual and compared to the true value and the point estimate from the confusion matrices (Figure S3).

REFERENCES

Fisher, R. (1922). On the interpretation of $\chi 2$ from contingency tables, and the calculation of P. *J. R. Stat. Soc.*, 85(1):87–94.

Gelman, A. (2003). A Bayesian Formulation of Exploratory Data Analysis and Goodness-of-fit Testing*. *Int. Stat. Rev.*, 71(2):369–382.

McElreath, R. (2018). *Statistical rethinking: A Bayesian course with examples in R and Stan*. Chapman and Hall/CRC.

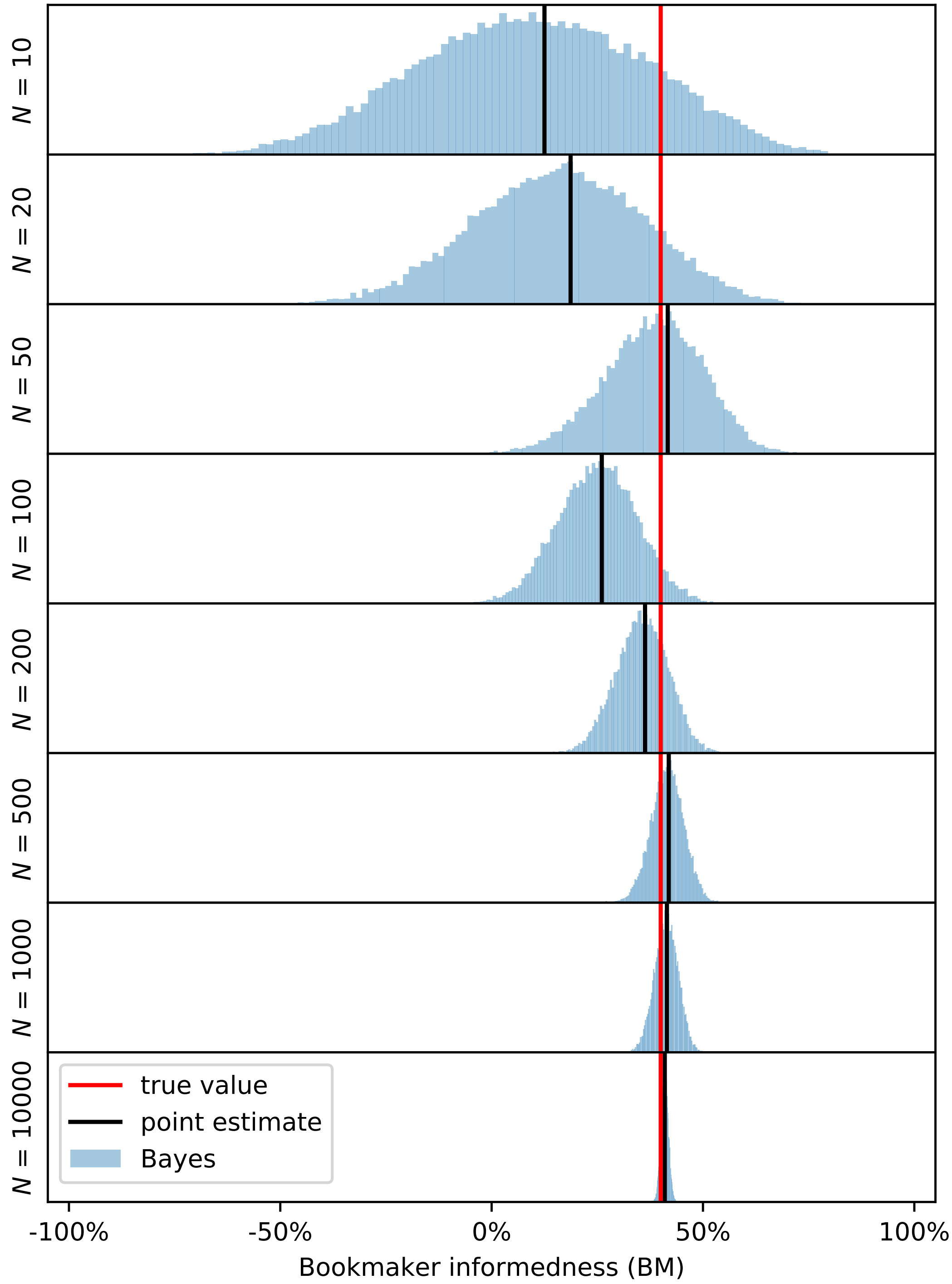

Figure S3. Metric uncertainty (MU) at varying sample sizes for a simulated classifier with known properties
